# The Involvement of *MGF505* Genes in the Long-Term Persistence of the African Swine Fever Virus in Gastropods

**DOI:** 10.3390/v17060824

**Published:** 2025-06-07

**Authors:** Sona Hakobyan, Nane Bayramyan, Zaven Karalyan, Roza Izmailyan, Aida Avetisyan, Arpine Poghosyan, Elina Arakelova, Tigranuhi Vardanyan, Hranush Avagyan

**Affiliations:** 1 Viral Ecology Research Group, Institute of Molecular Biology of NAS RA, E. Hasratyan 7, Yerevan 0014, Armenia; 2 Laboratory of Cell Biology and Virology, Institute of Molecular Biology of NAS RA, E. Hasratyan 7, Yerevan 0014, Armenia; 3 Laboratory of Antiviral Drug Discovery, Institute of Molecular Biology of NAS RA, E. Hasratyan 7, Yerevan 0014, Armenia; 4 Experimental Laboratory, Yerevan State Medical University, Yerevan 0025, Armenia; 5 Scientific Center for Risk Assessment and Analysis in Food Safety Area, 107/2 Masis Highway, Yerevan 0071, Armenia

**Keywords:** African swine fever virus, gastropods, atypical hosts, virus stability, host defence mechanisms, interferon (IFN) response, antiviral response, *MGF505* gene family, invertebrate reservoirs, virus–host interactions, evolutionary adaptation, viral evasion of immunity

## Abstract

African swine fever virus (ASFV), a highly contagious and lethal virus affecting domestic and wild pigs, has raised global concerns due to its continued spread across Europe and Asia. While traditional transmission pathways involve suids and soft ticks, this study investigates the potential role of freshwater gastropods as environmental reservoirs capable of sustaining ASFV. We analysed ASFV survival in ten gastropod species after long-term co-incubation with the virus. Viral transcriptional activity, particularly of the late gene *B646L* and members of the multigene family *MGF505*, was evaluated in snail faeces up to nine weeks post-infection. Results revealed that several gastropods, including *Melanoides tuberculata*, *Tarebia granifera*, *Physa fontinalis*, and *Pomacea bridgesii*, support long-term persistence of ASFV, accompanied by increased *MGF505* gene expression. Notably, the simultaneous activation of *MGF5052R* and *MGF50511R* significantly correlated with higher *B646L* expression and extended viral survival, suggesting a functional role in ASFV maintenance. Conversely, antiviral (AV) activity assays showed that some gastropod faeces reduced replication of the unrelated Influenza virus, hinting at induced host defences. A negative correlation was observed between AV activity and the expression of *MGF505 2R/11R*, implying that ASFV may suppress antiviral responses to facilitate persistence. These findings suggest that certain gastropods may serve as overlooked environmental hosts, contributing to ASFV epidemiology via long term viral shedding. Further research is needed to clarify the mechanisms underlying ASFV–host interactions and to assess the ecological and epidemiological implications of gastropods in ASFV transmission cycles.

## 1. Introduction

African swine fever virus (ASFV) is a DNA virus belonging to the *Asfarviridae* family, which causes a fatal disease in Eurasian pigs. In natural foci of the virus’s distribution across a large region of East, South, and South-Central Africa, the virus circulates between *Ornithodoros moubata* ticks and virus-resistant wild species of endemic suidae. Although no reservoirs or biological vectors have been identified in Eurasia, the virus has been shown to survive for extended periods and has demonstrated one of the most successful expansions [1]. It is well known that the large double-stranded DNA genome of the mature virus particle is encased in a membrane, which helps to maintain the stability of ASFV and its genome in most natural environments [2]. Also, ASFV exhibits a wide range of persistence depending on factors such as pH, ambient temperature, and the nature of the viral unusual host [3,4,5].

We consider that one potential factor contributing to this phenomenon is the virus’s ability to persist in various invertebrates, including gastropods [6,7]. Our previous work demonstrated that the virus does not actively replicate in gastropods, as indicated by a decrease in viral titers. However, the virus persists long-term in gastropods, and the expression of early, intermediate, and late viral genes was observed in some gastropod species [7]. These findings suggest that the long-term persistence of the virus may be attributed to alternative patterns of viral gene expression. Nevertheless, the mechanisms underlying the long-term persistence of the virus in gastropods remain unclear.

Upon viral invasion, complex multicellular organisms typically initiate an antiviral response.

Being one of the most important antiviral systems in vertebrates, the presence of interferon (IFN) system-mediated non-specific antiviral responses has also been gradually confirmed in invertebrates, supported by the identification of IFN-like components. These components, which include *IFN-like molecules, pattern recognition receptors (PRRs), IFN regulatory factors (IRFs), IFN-like receptors, JAK/STATs*, and *IFN-stimulated genes (ISGs)*, appear to form an IFN-like regulatory network in mollusks [8]. Generally, the antiviral response in mollusks shows at least partial similarities to the vertebrate IFN pathway [9,10]. Recent studies have highlighted the importance of autophagy in the antiviral response of oysters, as well as the role of the RNA interference (RNAi) pathway and apoptosis in molluscan immunity. A mechanism for Poly(I·C)-induced antiviral priming has also been described in oysters, representing a novel preventative strategy in mollusks [11,12,13,14].

It should be noted, however, that the aforementioned studies pertain to viruses that are highly infectious in mollusks, which ultimately result in the destruction of the host organism.

The ASFV genome consists of a single, linear, covalently closed-ended dsDNA, approximately170 to 194 kb in length, and contains five different multigene families (*MGF110*, *MGF360*, *MGF530/505*, *MGF300*, and *MGF100*). These families are localised in regions close to the ends of the genome. Members of two of these families, *MGF360* and *MGF505/530*, have been shown to be virulence determinants for pigs, acting by inhibiting type I IFN activity [15]. The major differences in the lengths of ASFV genomes among different isolates are attributed to the gain or loss of MGF gene members, including *MGF505*. This family has 11 members, and different variants of the ASFV contain 8–10 genes of *MGF505* [16,17,18,19].

Most of the known *MGF505* genes are involved in suppressing the host’s immune system. The *MGF505 2R* gene plays a role in suppressing innate immunity. Thus, deletion of *MGF505 2R* leads to the expression of genes associated with IFN synthesis and those related to inflammation and innate immunity. Refs. [20,21] showed that *MGF505 4R* interferes with the induction of the IFN-α/β pathway, through interaction with tumour necrosis factor receptor-associated factor 3. The ASFV *MGF505 7R* protein disrupts IFN-β signalling by interacting with IFN-regulatory factor 9. Additionally, *MGF505 11R* may inhibit cGAS-STING-mediated activation of IFN-β promoters and affect the expression of the stimulator of IFN genes (STING) through lysosomal, ubiquitin-proteasome, and autophagy pathways [22,23]. The aim of this study is to investigate whether ASFV induces an antiviral response during its long-term persistence in the gastropod host. Given the known functional involvement of *MGF505* genes in susceptible hosts, we also seek to elucidate the role of this multigene family in mediating virus–gastropod interaction.

## 2. Methods

### 2.1. Virus

Armenia 08 strain of the ASFV was used in the experiments [24]. This strain was first isolated in 2007 from the spleen of a swine infected with ASFV. The titration of the ASFV was conducted as previously described, expressed in hemadsorption units (HADU) as log10 HADU50/mL for non-adapted cells [25,26,27]. Additionally, the number of virus genome copies was quantified using qRT-PCR. The infection dose of the virus was determined by two factors: the virus concentration in water at 10^3^ HADU/mL and the body weight of the snails at 10^3^ HADU/gram (this latter parameter may have varied slightly due to challenges in accurately determining the ratio of mineral shell mass to body mass). The virus was subsequently added to all nine aquariums, in accordance with the specified conditions.

### 2.2. Sampling

All the nine species of chosen gastropods were kept in nine separate aquariums. The experimental part of the work was described earlier [7]. Briefly, all investigated snails were kept under a 12 h/12 h light/dark regime. The stock and experimental tanks were kept in a climate room at 25 °C (± 2 °C). Water tanks were covered to prevent evaporation. Water was changed 10% every four weeks to ensure good water quality. In the pre-experimental period (1 month), snails were kept under the same conditions to adapt and exclude bacterial diseases. Gastropod faeces were collected every week after the start of the experiment using a sterile pipette. After collection, the samples were centrifuged at 2000 rpm, the water was removed and the sediment was frozen at −30 °C. The gastropods were frozen in equal quantities at 4, 12, and 20 weeks after the start of the experiment. Measurements were performed 4 weeks after the start of the experiment. All the nine species of chosen gastropods were kept in nine separate aquariums. The experimental part of the work was described earlier [7].

### 2.3. Experimental ASFV Infection in Freshwater Gastropods

The ASFV (Armenia08) strain was used in all experiments in a dose in water 10^3^ HADU/mL. The gastropods were frozen in equal amounts at 4, 12, and 20 weeks since the beginning of the experiment. Measurements were taken 4 weeks after the start of the experiment. For the virus hemadsorption assay, inactivated ASFV served as a negative control. The virus was inactivated in a water bath at 65 °C for 10 min. After heat inactivation, the virus was tested for infectivity on porcine alveolar macrophages (PAMs) using the hemadsorption assay.

### 2.4. Quantitative Real-Time Polymerase Chain Reaction

Total viral RNA/DNA was extracted using the Viral RNA/DNA Prep kit (BIOFACT, Yuseong-gu, Daejeon, Republic of Korea). Here and below, all cDNA samples were treated with DNAse (Invitrogen 15657708) according to manufacturer instructions. For gastropod genes the RNA/DNA samples were subsequently reverse transcribed with the FIREScript^®^ RT cDNA synthesis kit (Solis Biodyne, Tartu, Estonia), following the manufacturer’s guidelines. The concentrations of the DNA/RNA were measured with a NanoDrop^®^ ND-1000 UV-Vis Spectrophotometer (Waltham, MA, USA), which included A260/280 ratio measurements (approximately 1.8 for DNA and around 2.0 for RNA). Quantitative Real-time polymerase chain reaction (qRT-PCR) was conducted using the SYBR green method, as previously described [28,29] on a Bio-Rad CFX 96 Real-Time PCR system (Bio-Rad Laboratories, Inc., Hercules, CA, USA). Each 20 µL reaction mixture contained 4 µL of 5× HOT FIREPol^®^ EvaGreen^®^ qPCR Mix Plus (ROX) (Solis BioDyne, Tartu, Estonia), 0.3 µL of each specific primer (initial concentration of 100 pmoL/µL), 4 µL of template DNA/cDNA, and 15.4 µL of ddH2O. DNA from the spleen of an ASFV-infected pig served as a positive control, while the water of gastropods’ vital activity without virus/uninfected water acted as a negative control. The standard curve method was used. The reaction conditions were as follows: polymerase activation at 95 °C for 12 min, followed by 40 cycles of 95 °C for 15 s, 52 °C for 30 s, and 72 °C for 30 s; and Melt Curve analyses were used under the following conditions: 65 °C to 95.0 °C: Increment 0.5 °C 0:05 s. Standard curves were generated using serial 10-fold dilutions of viral DNA. The fluorescence threshold value (Ct) was determined using CFX Maestro Software version 2.3 (Bio-Rad Laboratories, Inc., Hercules, CA, USA). Quantitative analysis of DNA/cDNA copies was performed by comparison with known numbers of ASFV genome units (standard log dilutions). The viral housekeeping genes *K196R* (Thymidine kinase gene) and *R298L* (Serine/threonine protein kinase gene) were used as reference genes. Primers used for amplification were designed based on ASFV Georgia 2007/1 sequence (Gene bank: FR682468.2), Physella acuta genes *Jak-1 like*, *STAT-1 like*, and *PKR* (NW_026732831.1, NW_026732338.1, NW_026732057.1) in FASTA format and were ordered from Integrated DNA Technology-IDT (accessed on 11 May 2022) [30]. For ease of perception, the data is presented in a rescaled form.

All primers are listed in Table 1.

### 2.5. Evaluation of IFN-like Activity in Snail Faeces

MDCK (Madin-Darby canine kidney) cells were propagated in Minimum Essential Medium (MEM) (Thermo Fisher, Grand Island, New York, USA), supplemented with 10% foetal bovine serum (FBS) and antibiotics. Stock of the Influenza H1N1 virus strain was amplified on MDCK cells, titrated, and stored at −80 °C [31]. To check whether or not samples harvested from snail faeces have any protection effect against virus infection, we also performed the plaque assay with the Influenza virus, H1N1 strain, in the presence or absence of indicated snail samples. Untreated sample (control) or samples were pretreated with Oseltamivire (5 µmol) as an influenza virus inhibitory drug or with snail faeces samples for 30 min, after which equal amount of virus (MOI of 0.001) was added to all wells after 1 h of virus adsorption inoculum was removed, and unbound viruses were washed once with fresh medium. Infected cells were covered with the 1% agarose overlay equally mixed with 2 × infection media containing with/without 10% snail samples described elsewhere above. At 2 days, post-infection cells were fixed with 10% formaldehyde, stained with crystal violet, and photographed.

### 2.6. Study of Double-Stranded RNA in Physella Acuta Snail

Double-stranded RNAs (dsRNAs) were generated by in vitro transcription as previously described [32]. Briefly, single-stranded RNAs (ssRNAs) were transcribed in vitro from linearized plasmid constructs using T3 and T7 phage RNA polymerases (Sigma-Aldrich, St. Louis, Missouri, USA, RPOLT7-RO). The DNA template was subsequently degraded by the addition of DNase I (Sigma-Aldrich, 11284932001) at a ratio of 1 U/μg of DNA. Complementary RNA strands were mixed in a solution containing 400 mM of NaCl and 10 mM of Tris-Cl (pH 7.4), and annealed through a stepwise incubation at 75 °C for 15 min, 65 °C for 15 min, and room temperature for 15 min. Successful dsRNA formation was verified by size shift analysis using agarose gel electrophoresis. The concentration of dsRNA was determined spectrophotometrically using a NanoDrop instrument.

Before the experiment, the effect of different doses of dsRNA on the survival of snails was analysed (Figure S1). Taking into account the experimental data, a dose was selected that did not cause a lethal effect for 96 h after application. So, for experimental applications, dsRNA was administered at a concentration of 10 μg per gram of Physella acuta body weight (average snail weight: 31 mg). The conditions of keeping snails for the dsRNA experiment were carried out similarly to the gene expression experiments. Gene expression studies in studying the effect of dsRNA were carried out after 72 h of application. All snails were divided into 4 groups: 1st intact group (n = 10); 2nd group with inactivated virus (n = 20); 3rd group with active ASFV (n = 20); 4th group with dsRNA (n = 20).

After 24 h, ASFV (in the same doses as for the main experiment) was added, and the transcription of corresponding genes was analysed.

### 2.7. Statistical Analysis

Statistical analyses were performed using Student’s *t*-test, and by non-parametric Mann–Whitney U test. Spearman’s correlation was performed to determine whether there was a relationship between the AV effect, the expression of both *MGF505* 2R and *11R*, and *B656L* as well as long-term persistence data.

## 3. Results

### 3.1. Survival of ASFV After Coincubation in Gastropods

**Figure 1:**
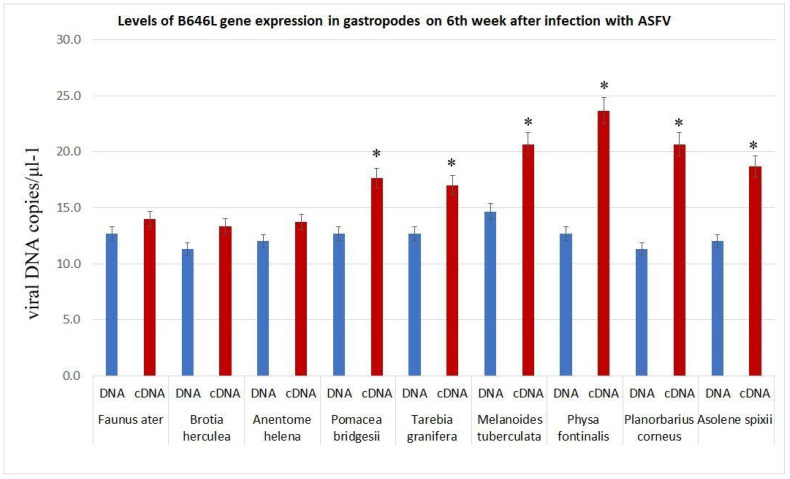
Expression of ASFV B646L gene in feaces of gastropods 6 weeks post infection. The data is presented in a rescaled form. * significant (*p* < 0.05) compared to DNA levels.

To analyse the capacity of long-term persistence of ASFV within gastropods, we examined the transcriptional activity of the *B646L* gene. The selection of the *B646L* gene is based on its importance in the virus replication cycle [33] and its classification as a late viral gene. As shown in Figure 1, transcriptional activity of the *B646L* gene is observed in the faeces of snails capable of supporting the long-term persistence of the ASFV. The exception is *Planorbarius corneus*, which does not support long-term persistence of the virus; however, transcriptional activity of the B646L gene is detected in its faeces (see below).

### 3.2. ASFV Surviving in Gastropods and Activity of MGF505

**Figure 2:**
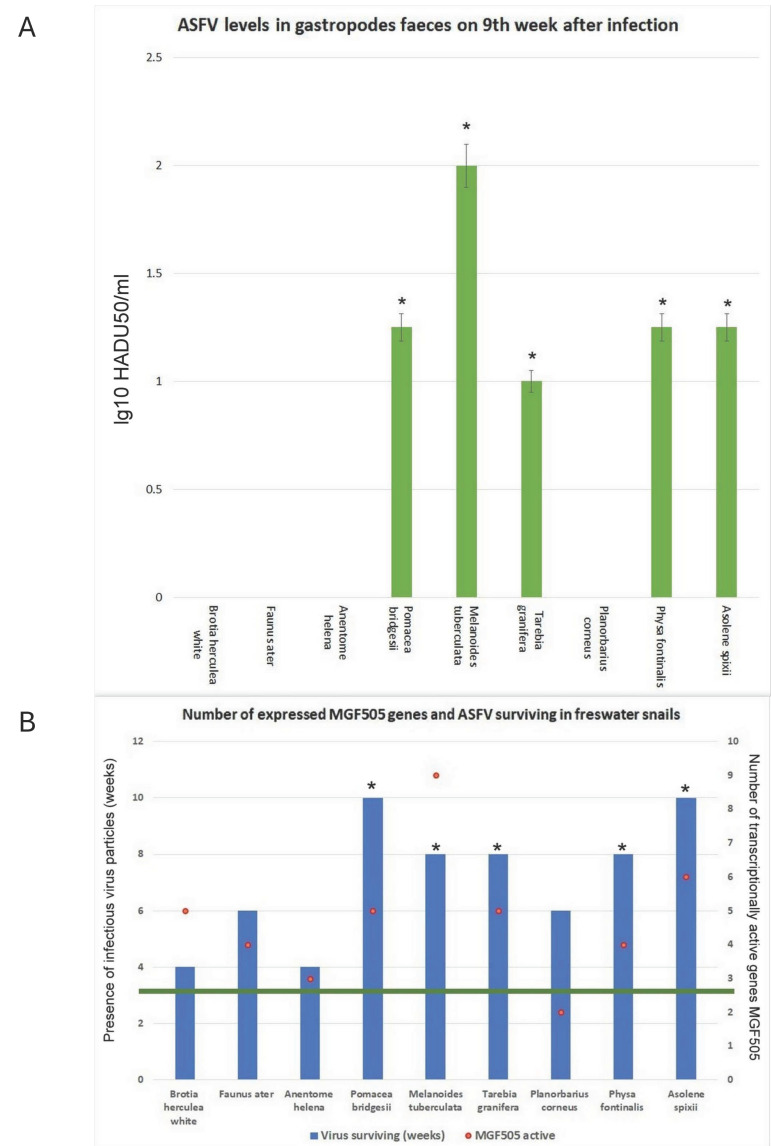
ASFV levels in gastropod faeces 9 weeks post-infection. (**A**). Significant data compared to levels in *Brotia herculea* and *Anentome Helena* (*p* < 0.05) (mean ± SD). (**B**). Number of activated genes from the *MGF505* family and ASFV survival time in gastropod faeces. The green line indicates the maximum time for the detection of infectious ASFV particles in water. * Significant compared to ASFV survival time in water (*p* < 0.05).

Previous studies have shown that the virus can survive for a long time in freshwater gastropods, in contrast to similar conditions in water, where it typically survives for no more than 2–3 weeks [7]. Figure 2A presents the infectious titers of the ASFV in gastropod faeces after 9 weeks of co-incubation with snails. From Figure 2A, it is evident that *Brotia herculean*, *Faunus ater*, *Anentome helena*, and *Planorbarius corneus* do not support the long-term survival of the ASFV.

Figure 2B displays data on the number of active genes within the *MGF505* family and the duration of virus survival in the presence of gastropods. Certain gastropods maintain infectious ASFV particles significantly longer than the control group. The same snails that sustain high infectious titers also demonstrate a greater ability to persist over time. As shown in Figure 2B, different gastropod species exhibit varying levels of *MGF505* gene activity. The number of expressible *MGF505* genes ranges from two in *Planorbarius corneus* to nine in *Melanoides tuberculata*. Analysis of *MGF505* gene expression revealed an increase in the number of active genes in gastropods that support longer virus survival periods (Figure 2B).

### 3.3. Transcription Pattern of MGF505 Genes

**Figure 3:**
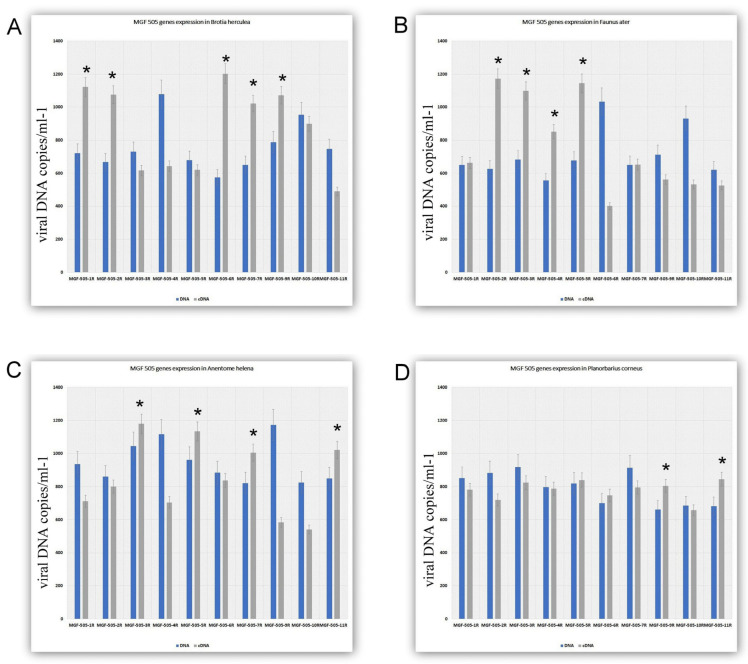
The expression of ASFV MGF genes 5 weeks after infection in gastropods. (**A**). MGF gene expression in *Brotia herculean*. (**B**). MGF gene expression in *Faunus ater*. (**C**). MGF gene expression in *Anentome helena*. (**D**). MGF gene expression in *Planorbarius corneus*. The data is presented in a rescaled form. * Significant (*p* < 0.05) compared to DNA levels.

The *MGF505* family in ASFVs of genotype 2 (Arm008) comprises 10 genes. We investigated the expression of all *MGF505* genes in the gastropods studied in the article [7]. Based on the data regarding virus load in the faeces of the gastropods, we categorised all snails into two groups: those that are unable to support the long-term survival of ASFV (Figure 3) and those that reliably support a longer duration of the virus (Figure 3). As illustrated in Figure 2, the ASFV is capable of expressing several genes from the MGF family in most snails. In the snail *Brotia herculea*, 5 out of 10 genes in the *MGF505* family are active. The transcriptionally active genes in *Brotia herculea* are *MGF505 1R*, *2R*, *6R*, *7R*, and *9R* (Figure 3A). In *Anentome helena* and *Faunus ater*, 4 out of 10 genes in the *MGF505* family are active. The transcriptionally active genes in *Faunus ater* are *MGF505 2R*, *3R*, *4R*, and *5R* (Figure 3B), while the active genes in *Anentome helena* are *MGF505 3R*, *5R*, *7R*, and *11R* (Figure 3C). The only exception is *Planorbarius corneus*, in which the expression of only two genes is detected (Figure 3D). The transcriptionally active genes in *Planorbarius corneus* are *MGF505 9R* and *11R* (Figure 3D).

**Figure 4:**
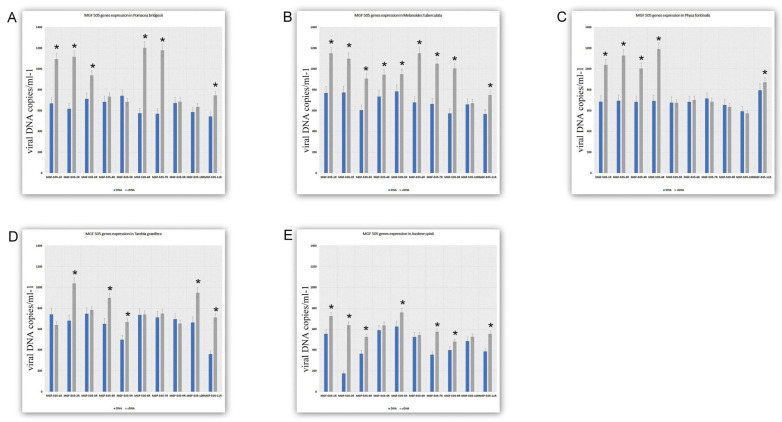
The expression of ASFV MGF genes 5 weeks after infection in gastropods. (**A**). MGF gene expression in *Pomacea bridgesii*. (**B**). MGF gene expression in *Melanoides tuberculata* (**C**). MGF gene expression in *Physa fontinalis* (**D**). MGF gene expression in *Tarebia granifera*. (**E**). MGF gene expression in *Asolene spixii.* The data is presented in a rescaled form. * Significant (*p* < 0.05) compared to DNA levels.

Figure 4 presents data on the transcriptional activity of the *MGF505* genes in gastropods capable of sustaining the long-term presence of ASFV. As shown in Figure 4, the number of transcriptionally active genes in snails from this group ranges from 5 to 9 out of 10. In *Pomacea bridgesii*, the transcriptionally active genes are *MGF505* 1R, 2R, 3R, 6R, 7R, and *11R* (Figure 4A). In *Melanoides tuberculata*, all genes are active, except for *MGF505 10R* (Figure 4B). The transcriptionally active genes in *Physa fontinalis* are *MGF505 1R*, *2R*, *3R*, *4R*, and *11R* (Figure 4C). In *Tarebia granifera*, the active genes are *MGF505 2R*, *4R*, *5R*, *10R*, and *11R* (Figure 4D). Finally, in *Asolene spixii*, the transcriptionally active genes include *MGF505 1R*, *2R*, *3R*, *5R*, *7R*, *9R*, and *11R* (Figure 4E).

**Figure 5:**
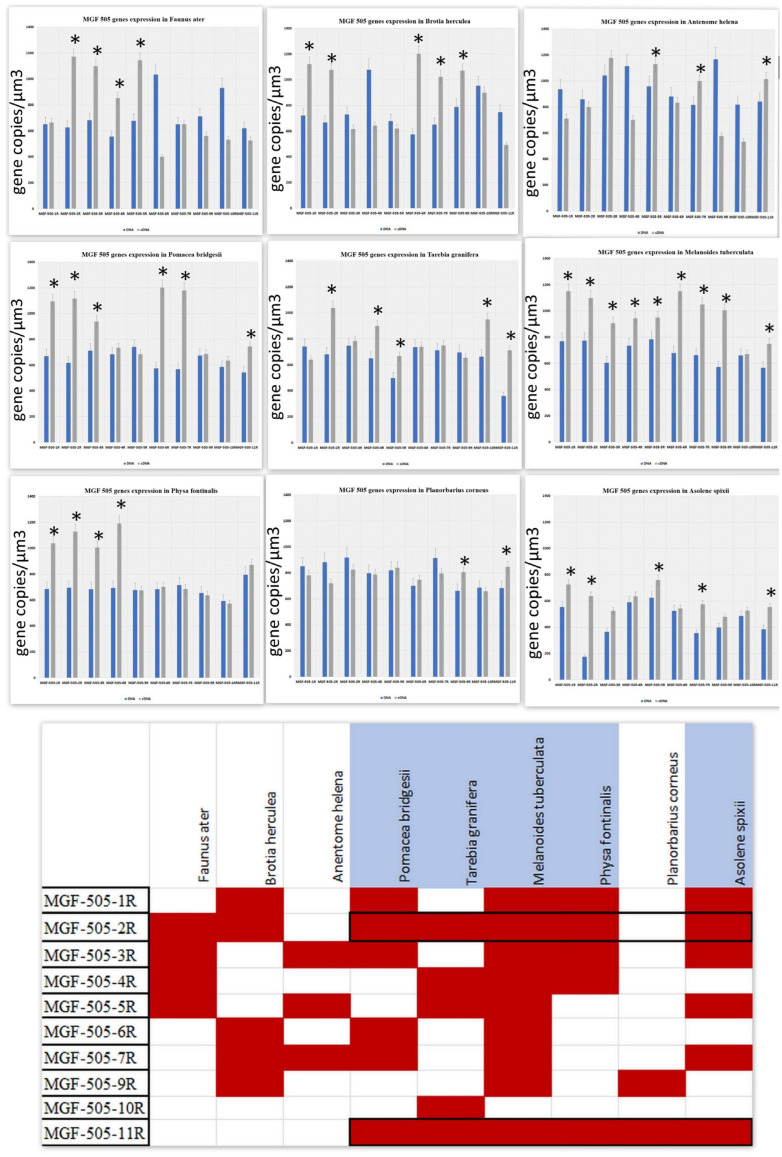
Analysis of the relationship between ASFV survival and *MGF505* family genes expression. The data is presented in a rescaled form. * Significant (*p* < 0.05) compared to DNA levels. In the scheme, the data for the snails in which the virus survived significantly longer compared to the control (ASFV in water) are highlighted in blue.

Analysis of the relationship between ASFV survival and the activity of the *MGF505* family genes revealed a tendency (though not statistically significant) for increased virus survival time correlating with the number of active genes (Figure 5). Figure 5 shows confluent monolayers of MDCK cells in 24-well tissue culture plates. Notably, all gastropods capable of long-term support of infectious ASFV titers exhibited transcriptional activity of the *MGF505 2R* and *11R* genes (Figure 5).

### 3.4. ASFV Evasion of Antiviral Responses in Gastropods

The next step in our research was to investigate the antiviral activity of the studied gastropods. It should be noted that the absence of reference genome sequences for the freshwater gastropod species used in this study has limited the application of molecular genetics and bioinformatics approaches to investigate the immune response in gastropods to ASFV infection. Antiviral activity was examined in gastropod faeces, considering previous studies [7] that reported high levels of ASFV accumulation in the snail gut. Such viral levels were expected to potentially induce a host antiviral response.

**Figure 6:**
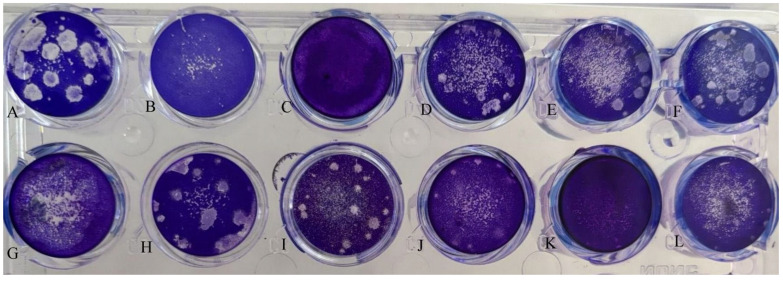
Inhibition of the replication of H1N1 influenza virus in MDCK cells by homogenates of snails faeces. Bright-field images of crystal violet-stained MDCK cells infected with Influenza H1N1 virus (48 h post infection). Cultures were infected with viruses at an MOI of 0.01 PFU/cell. Virus titers were determined as log10 PFU/mL. (**A**) Virus control; (**B**) Protective AV action of the faeces of *Faunus ater* infected with the ASFV; (**C**) Protective AV action of the faeces of *Brotia herculea* infected with the ASFV; (**D**) Protective AV action of the faeces of *Anentome helena* infected with the ASFV; (**E**) Protective AV action of the faeces of *Pomacea bridgesii* infected with the ASFV; (**F**) Protective AV action of the faeces of *Melanoides tuberculata* infected with the ASFV; (**G**) Protective AV action of the faeces of *Planorbarius corneus* infected with the ASFV; (**H**) Protective AV action of the faeces of *Tarebia granifera* infected with the ASFV; (**I**) Protective AV action of the faeces of *Physa fontinalis* infected with the ASFV; (**J**) Protective AV action of the faeces of *Asolene spixii* infected with the ASFV; (**K**) MDCK cells control; (**L**) Protective AV action of the oseltamivir.

To evaluate the qualitative value of virus reduction (protective response), we used homogenates from all snail faeces and tested them against the Influenza virus, as described above. Figure 6 shows that in the presence of 10% collected samples, there was a significant reduction in viral plaque formation compared to the virus control (Figure 6) and samples lacking *MGF505* gene expression. For the Influenza virus inhibitory reference control, we also used Oseltamivir at a concentration of 5µM [33].

As shown on Figure 6 in the control infection of the virus, multi-membered plaques are visible (Figure 6A). Faeces of *Faunus ater* and *Brotia herculea* (Figure 6B,C) have a protective effect and block the formation of viral plaques. Faeces of *Anentome Helena* (Figure 6D), *Pomacea bridgesii* (Figure 6E), *Melanoides tuberculata* (Figure 6F), *Planorbarius corneus* (Figure 6B,G), *Tarebia granifera* (Figure 6H), *Physa fontinalis* (Figure 5I), and *Asolene spixii* (Figure 6J) do not have a protective effect (*Planorbarius corneus* partially), data on MDCK cells control (Figure 6K) and Protective AV action of the Oseltamivir (Figure 6L) are also given. The results correspond with genes’ activation of the MGF-505 family. The only exception is *Anentome helena.*

As a complementary approach to the method described above, we conducted a transcriptional analysis of tyrosine-protein kinase JAK1-like, signal transducer and activator of transcription STAT1-like, and IFN-induced, double-stranded, RNA-activated protein kinase (PKR)-like genes in Physella acuta, a gastropod species closely related to Physa fontinalis, under the influence of double-stranded RNA (dsRNA). To assess the impact of ASFV, the expression of the same genes was also examined following viral exposure. The results indicate that dsRNA treatment induced the expression of JAK1-like and STAT1-like genes; however, it does not affect PKR-like genes in Physella acuta, whereas this antiviral response was markedly suppressed in the presence of active ASFV (Supplementary Material S1, Figures S2–S4).

Table 2 presents correlation data between the activation of the *MGF505 2R* and *MGF505 11R* genes and the expression of the *B646L* gene, which encodes the structural protein p72, as well as their association with antiviral (AV) effects. In addition, a correlation analysis was performed between *MGF505 2R* and *11R* activation and long-term viral persistence.

As shown in Table 2, the simultaneous expression of the *MGF505 2R* and *11R* genes significantly correlates with the expression level of the *B656L* gene and the long-term survival of ASFV in the snail gut. A negative correlation was also observed between the antiviral effect of snail faeces and the simultaneous expression of the *MGF505 2R* and *11R* genes. Our studies demonstrate that the antiviral activity in snail faeces is induced by the presence of ASFV, likely contributing to the relatively rapid elimination of the virus from the snails’ bodies. Furthermore, the simultaneous expression of the *MGF505 2R* and *11R* genes results in an increase in *B656L* gene expression. Additionally, we found that the extended survival of ASFV occurs only in those snails where simultaneous expression of the *MGF505 2R* and *11R* genes is detected.

## 4. Discussion

ASFV is a unique and complex pathogen that is maintained in sub-Saharan Africa through a sylvatic cycle involving wild suids such as warthogs, bushpigs, and soft ticks of the genus Ornithodoros. Since 2007, ASFV, particularly genotype II, has spread to the Caucasus region and, since 2014, to various parts of Europe. The epidemiology of ASFV in Eurasia suggests the involvement of alternative transmission pathways, underscoring the need for continued scientific investigation to elucidate the complexities of viral dissemination. Previous studies have demonstrated ASFV’s ability to infect an atypical invertebrate host. In this context, arthropods commonly found in the Eurasian region such as ticks, mosquitoes, and lice have been considered as potential vectors or reservoirs for viral persistence and transmission [34,35,36,37,38]. In general, there is a lack of evidence supporting the existence of long-term invertebrate carriers of ASFV except soft ticks of the genus Ornithodoros [39]. Moreover, transcriptomic analyses that could elucidate the molecular mechanisms underlying long-term viral persistence in atypical hosts are largely lacking.

Recently, ASFV has also been shown to replicate within leeches, where the virus likely undergoes an abortive replication cycle [35].

Our previous studies has demonstrated that ASFV is capable of surviving for prolonged periods in gastropods, which are considered atypical hosts for the virus [6,7].

Assessing ASFV’s ability to replicate within gastropods is challenging. On the one hand, long-term preservation of infectious titers (expressed in HADU) suggests the presence of intact viral particles. On the other hand, we found no reliable indicators of growth in this parameter. We identified the transcriptional activity of the capsid gene *B646L*, a late viral gene that is essential for virus assembly. Its activation is observed in gastropods capable of maintaining the virus’s survival for an extended time (except for *Planorbarius corneus*). This observation, in conjunction with infectious titer data, may indirectly suggest the completion of a replication cycle for ASFV.

Notably, gastropods have been incubated with ASFV at temperatures ranging from 22 to 24 °C. Temperature is known to affect all stages of viral replication, including cell entry, genome replication, assembly, release of new virions, and the development of a virus-induced pathology. While this phenomenon has been studied more extensively in RNA viruses [39], it is also highly relevant to DNA viruses. In mollusks infected with DNA viruses, for instance, pathological changes associated with elevated temperatures have been reported [40]. Temperature plays a particularly critical role in the biology of arboviruses, including ASFV, which is classified as a DNA arbovirus. Arboviruses typically follow a life cycle that involves replication in both a vertebrate host—with a stable internal temperature—and an arthropod vector, which lacks such thermal stability. The effect of temperature on arbovirus replication is twofold: elevated temperatures appear to enhance viral replication, while reduced temperatures can suppress the host’s antiviral immune response [41]. The replication cycle may slow significantly at lower temperatures—by several orders of magnitude [42]. Given these factors, the hypothesis of a unique, temperature-dependent cascade regulation of ASFV replication—potentially influenced by the atypical nature of the gastropod host—cannot be excluded.

However, the expression of specific early, ambivalent, and late viral genes in these organisms suggests the existence of an alternative mechanism for viral persistence/survival [7]. Once the virus has entered the cells of a non-typical host, several factors may limit its replication. The stability of the virus within an atypical host can be attributed to the activation of genes that suppress the host’s defence mechanisms.

In this study, we sought to determine whether the virus, during its long-term persistence in gastropods, elicits any antiviral response.

Although the antiviral defence system in mollusks is insufficiently studied, several components of the (IFN)-like system have been identified and characterised, including IFN-like molecules, pattern recognition receptors, IFN regulatory factors, IFN-like receptors, suppressors of cytokine signalling, and IFN-stimulated genes. Many of these components share conserved structural and functional features with their vertebrate homologues. They can be activated by immune stimuli, particularly viral stimulation. However, these studies have primarily focused on the antiviral responses of molluscan species such as oysters and mussels, which are of significant economic and ecological importance worldwide, and are highly susceptible to viral infections [11,12,13,14]. Due to their well-characterised biological features revealed through genome sequencing, these species serve as valuable model organisms for studying antiviral responses. Previous research showed that a significant portion of the ASFV in gastropods is concentrated in the intestine [7]; thus, to study the virus’s effect on the IFN-like activity of snails, we prepared a faecal homogenate. We found that some gastropods exhibited antiviral activity in their faeces following ASFV infection. Then, we analysed the potential of ASFV to evade the IFN-like pathway in gastropods, thereby ensuring its long-term survival.

It has been previously reported that ASFV employs multiple self-encoded proteins to evade the host’s innate and adaptive immune responses. Suppression of the host IFN pathways is critical for successful ASFV replication, which likely explains why several key components of the IFN signalling cascade are targeted by various ASFV-encoded inhibitors. A primary role in this immune evasion process is attributed to members of the multigene families (*MGF*s) [43]. Most genes within the *MGF505* family are transcribed during the early phase of ASFV infection, classifying them as early genes, with the exception of *MGF505* 2R, which shows a different expression profile. Nearly all members of this gene family are implicated in the suppression of host immune signalling pathways and are involved in the host range [44,45]:

Studies examining the evolution of individual *MGF505* family genes have revealed relatively low genetic diversity, indicating a comparatively slow evolutionary rate for these genes [20].

Analysis of *MGF505* gene expression in various gastropods demonstrated a diverse activation pattern, ranging from the expression of only two genes in *Planorbarius corneus* to nine genes in *Melanoides tuberculata*. However, a comparison between the *MGF505* activation pattern and the ASFV survival time, *B656L* expression data in different snail species indicated that survival duration is influenced by the expression of two *MGF505 2R* and *11R* genes (Table 2). This expression is observed in all snails capable of maintaining the virus for extended periods, while it is absent in all others. It is likely that the expression of the remaining *MGF505* genes serves an auxiliary function.

Notably, the obtained data of antiviral activity (Figure 6) was shown only in gastropods in which the ASFV could not simultaneously activate both *MGF505 2R* and *11R* genes (Figure 5). It is important to note that, first, the faeces of control gastropods (not infected with the ASFV) did not demonstrate antiviral activity, and second, the activation of any other MGF genes (without simultaneous transcription) did not eliminate antiviral activity in the faeces of infected gastropods. Correlation of the activity data of these genes with antiviral response data (Table 2) provides preliminary evidence for their potential involvement in antiviral response evasion.

In addition to the antiviral findings mentioned above, we investigated the antiviral effect of ASFV in the gastropod Physella acuta, whose genome sequence is available. To stimulate the immune response, we used double-stranded RNA (dsRNA), a well-established inducer of cytokine and IFN activation cascades in vertebrates. In invertebrates, one of the known immune response mechanisms involves the interaction between dsRNA and the Janus kinase–signal transducer and activator of transcription (*JAK-STAT*) pathway. Based on this mechanism, synthetic dsRNA analogues, such as polyinosinic:polycytidylic acid (poly I:C), are currently being developed for antiviral protection in oysters [9,10,11,12]. Our results indicate that some genes typically activated by dsRNA stimulation were repressed 24 h post-ASFV infection.

While these findings suggest a potential suppression of the JAK-STAT pathway by ASFV, multigene family (*MGF*) genes—particularly members of the MGF360 and *MGF505* families—have been identified as major virulence factors associated with ASFV pathogenesis through their degrading *JAK/STAT* signalling pathway in the host [43,45,46]. Further investigation is required to validate and elucidate the underlying molecular mechanisms.

Previous studies have demonstrated that genes within the *MGF505* family contribute to ASFV’s ability to evade the host innate immune response, particularly by inhibiting type I IFN signalling pathways. This function is critical for efficient viral replication in porcine macrophages, the primary target cells in swine. Deletions or mutations within the *MGF505* region have been associated with attenuated viral phenotypes and a restricted host range, suggesting that *MGF505* may play a key role in determining host tropism. In the context of our findings, it is plausible that *MGF505* expression also influences ASFV persistence capacity in unusual hosts such as gastropods. Although the precise mechanisms remain to be elucidated, these observations support the hypothesis that *MGF505* contributes to virally adaptat across diverse host species.

## 5. Conclusions

The coordinated transcription of *MGF505 2R* and *MGF505 11R* is likely a key factor facilitating the long-term persistence of ASFV in freshwater snails. ASFV appears to be capable of both inducing and evading certain antiviral responses in gastropods.

## Figures and Tables

**Table 1:** Oligonucleotide primer details used in the qRT-PCR assay.

Gene	Sequence
*K196R*	F:GCAGTTGTCGTAGATGAAG R: 5′-CGAAGGAAGCATTGAGTC
R298L	F: 5′-TCTGAAATGTTCTCGGGAAT-3′ R: 5′-GTGTGGACGATAGGTATGG-3
*MGF505*-1R	F: ACGCACAGATAGAACAAT R: TGGCAACATAATGGCTTA
*MGF505*-2R	F: AGAGTGAACCTGATAGAT R: TAAGAAGTATGGATTACGATA
*MGF505*-3R	F: GGCTACTCAATTATCCTT R: GCTTCCACCATATTCTAT
*MGF505*-4R	F:AATATGGCAGTCTTATCTAA R:ATGGCGGTTAATAATAGGv
*MGF505*-5R	F:TGGAGAGGATATTCAAGT R:TAGATAATAAGGACACATTCA
*MGF505*-6R	F:GGATGTAACGATAATAGTCT R: CCAACAATAATAGTTCTTCAA
*MGF505*-7R	F:GAGGACTGAGAACTATAAC R: ATCACATTCAAGGCTTAA
*MGF505*-9R	F:TGCTATCAATCCATAAGG R:TGAATCAGTGGTAGAATC
*MGF505*-10R	F:AGGAGGTCTTCTTAACTT R:CACAGCATAGAGTAACAG
*MGF505*-11R	F:GCCAATAATCATCACAGA R: GTAATACACCGAATCAATG
B646L	F: CCGATCACATTACCTCTTATTAAAAACATTTCC R: GTGTCCCAACTAATATAAAATTCTCTTGCTC
*tyrosine-protein kinase JAK1-like*	F-GTTGCTAAGGTGTCAGATT R-CAGGTAATGTAGATTCAGGTT
*signal transducer and activator of transcription STAT1-like*	F-CCAAGTCATTCCAATAAGTAAT R-CTCTACATCAGCAATATCCA
*Physella acuta IFN-induced, double-stranded RNA-activated kinase-like protein*	F-AAGCAGAAGCCAGAGATG, R-ACCGATGTATATGAAGATAGTGTA

**Table 2:** The correlation data between the ASFV surviving, the AV effect, the expression of both *MGF505 2R* and *11R*, and the expression of *B656L*.

		Long-Term Survival	*MGF505 2R 11R* Activation	*B656L* Expression	AV Activity
**Long-term survival**	Correlation Coefficient	1	0.791 (**)	1.000 (**)	−0.5
	Sig. (1-tailed)	.	0.006	.	0.085
***MGF505* 2R 11R activation**	Correlation Coefficient	0.791 (**)	1	0.791 (**)	−0.632 (*)
	Sig. (1-tailed)	0.006	.	0.006	0.034
**B656L expression**	Correlation Coefficient	1.000 (**)	0.791 (**)	1	−0.5
	Sig. (1-tailed)	.	0.006	.	0.085
**AV activity**	Correlation Coefficient	−0.5	−0.632 (*)	−0,5	1
	Sig. (1-tailed)	0.085	0.034	0.085	.

* Correlation is significant at the 0.05 level (1-tailed). ** Correlation is significant at the 0.01 level.

## Data Availability

Data are available with this article.

## Supplementary Materials

The following supporting information can be downloaded at: https://www.mdpi.com/article/10.3390/v17060824/s1, Figure S1: Toxic action of dsRNA on survival of *Physella* acuta snail in 72 h post application; Figure S2: Effect of 10 µg of dsRNA per 1 g of *Physella* acuta snail weight on the expression of the Jak1-like gene; Figure S3: Effect of 10 µg of dsRNA per 1 g of *Physella* acuta snail weight on the expression of the STAT 1-like gene; Figure S4: Effect of 10 µg of dsRNA per 1 g of *Physella* acuta snail weight on the expression of the PKR-like gene.
