# Supplementary material for: The Involvement of MGF505 Genes in the Long-Term Persistence of the African Swine Fever Virus in Gastropods
Source: Viruses. 2025 Jun 7;17(6):824. doi: 10.3390/v17060824 (PMC12197388; doi:10.3390/v17060824)
Supplement: Supplementary file 1 [file viruses-17-00824-s001.zip › viruses-3666967-supplementary.pdf]

Figure S1. Toxic action of dsRNA on survival of *Physella acuta* snail in 72 h post application.

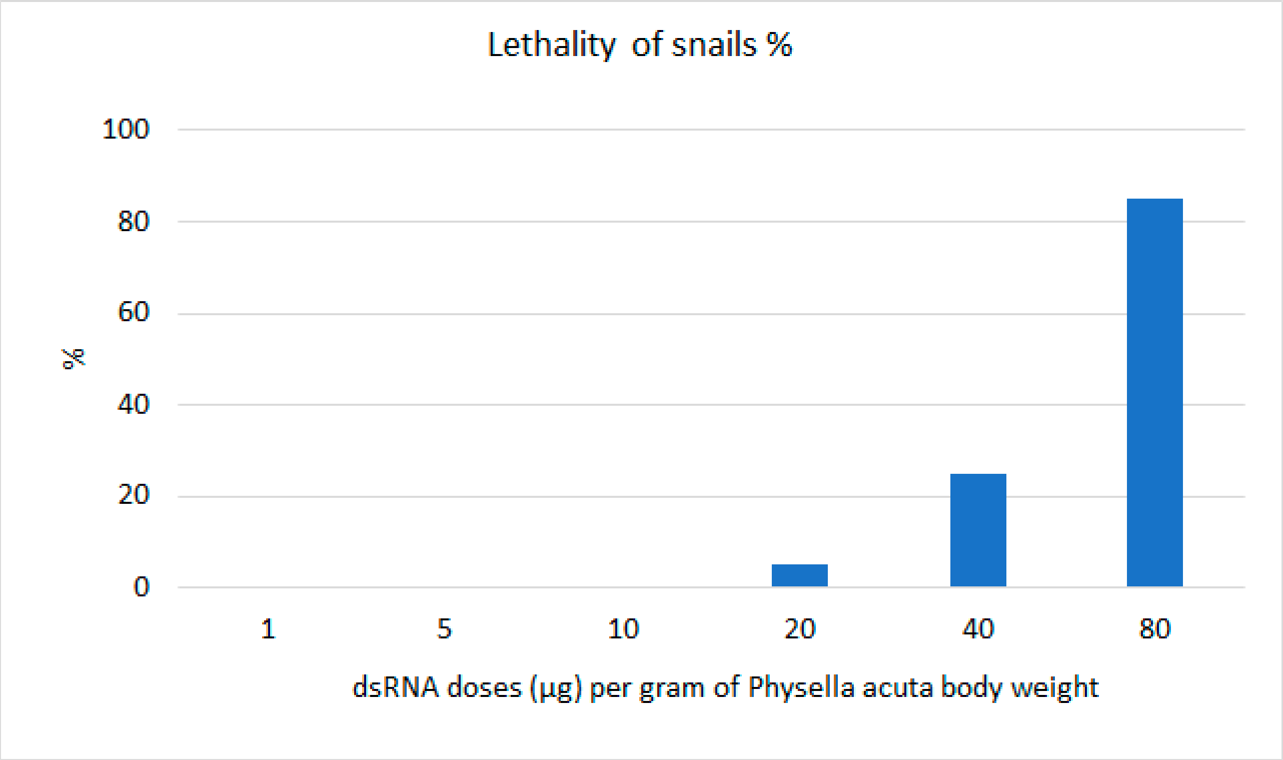

As follows from Figure S1, high doses of dsRNA are toxic to the *Physella acuta* snail. Already a dose of 20 µg per 1 g of snail weight resulted in insignificant mortality. Even a dose of 80 µg per 1 g of snail weight was lethal for almost 90% of snails within 72 h after dsRNA application.

Figure S2. Effect of 10 µg of dsRNA per 1 g of *Physella acuta* snail weight on the expression of the Jak1-like gene.

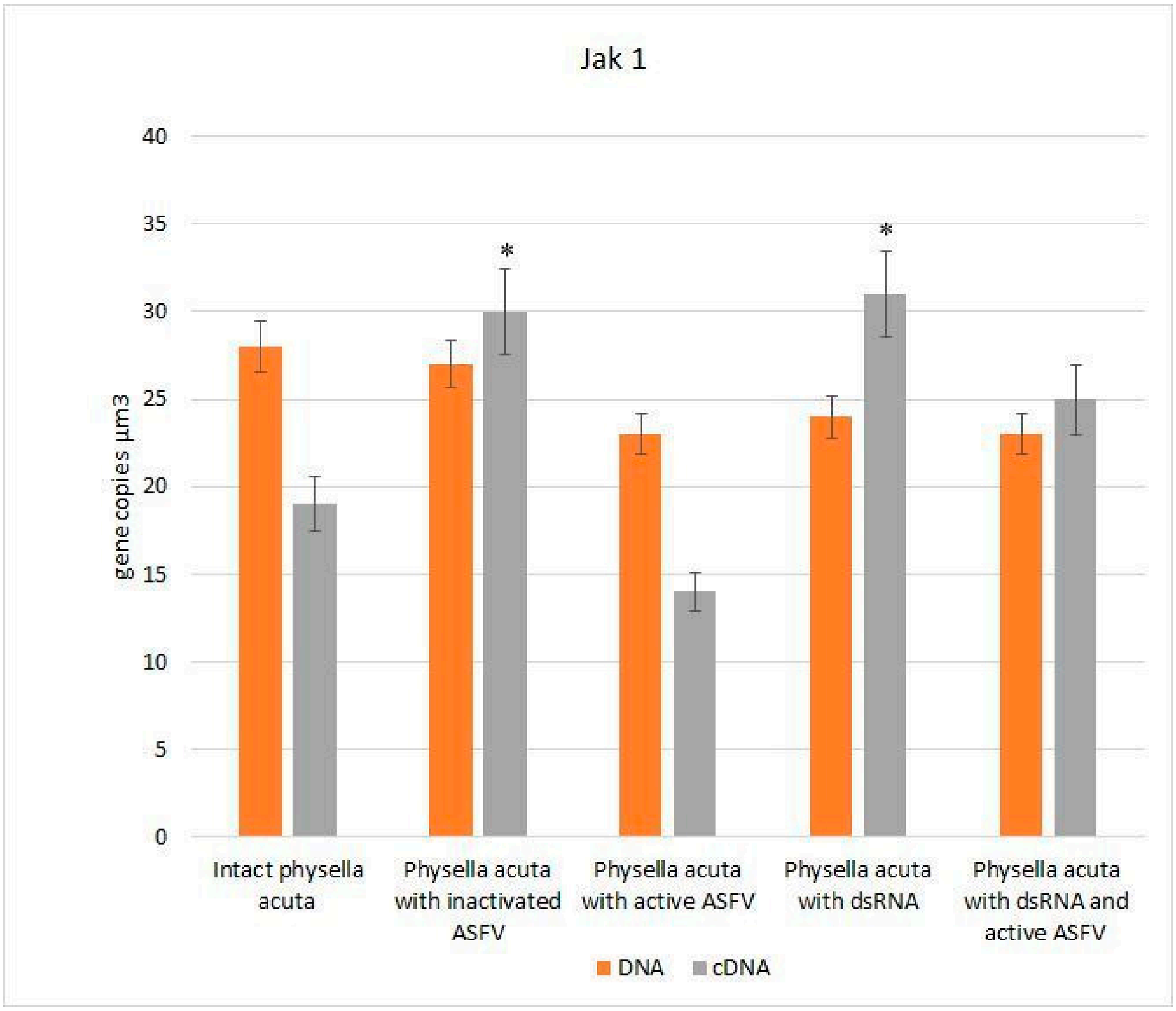

*Significant compared to intact *Physella acuta* with active ASFV ($p<0.05$).

As follows from Figure S2, inactivated ASFV and dsRNA are able to activate transcription of the Jak1-like gene in the *Physella acuta* snail. It should be noted that the infection of snails with active ASFV inhibits the transcriptional activity of this gene.

Figure S3. Effect of 10 µg of dsRNA per 1 g of *Physella acuta* snail weight on the expression of the STAT 1-like gene.

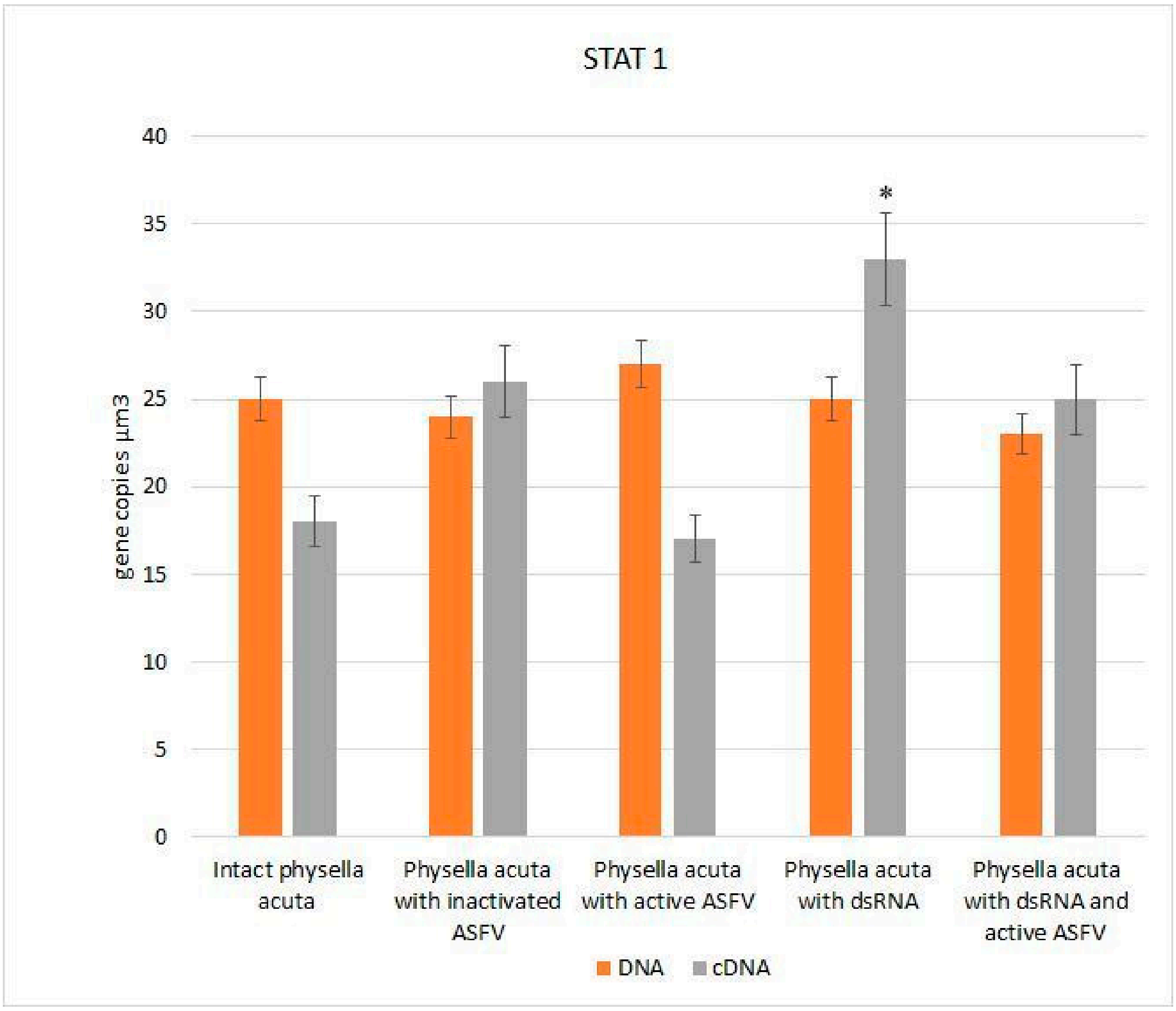

*Significant compared to intact *Physella acuta* with active ASFV ($p<0.05$).

As follows from Figure S3, dsRNA is able to activate transcription of the STAT 1-like gene in the *Physella acuta* snail. It should be noted that the infection of snails with active ASFV inhibits the transcriptional activity of this gene.

Figure S4. Effect of 10 µg of dsRNA per 1 g of *Physella acuta* snail weight on the expression of the PKR-like gene.

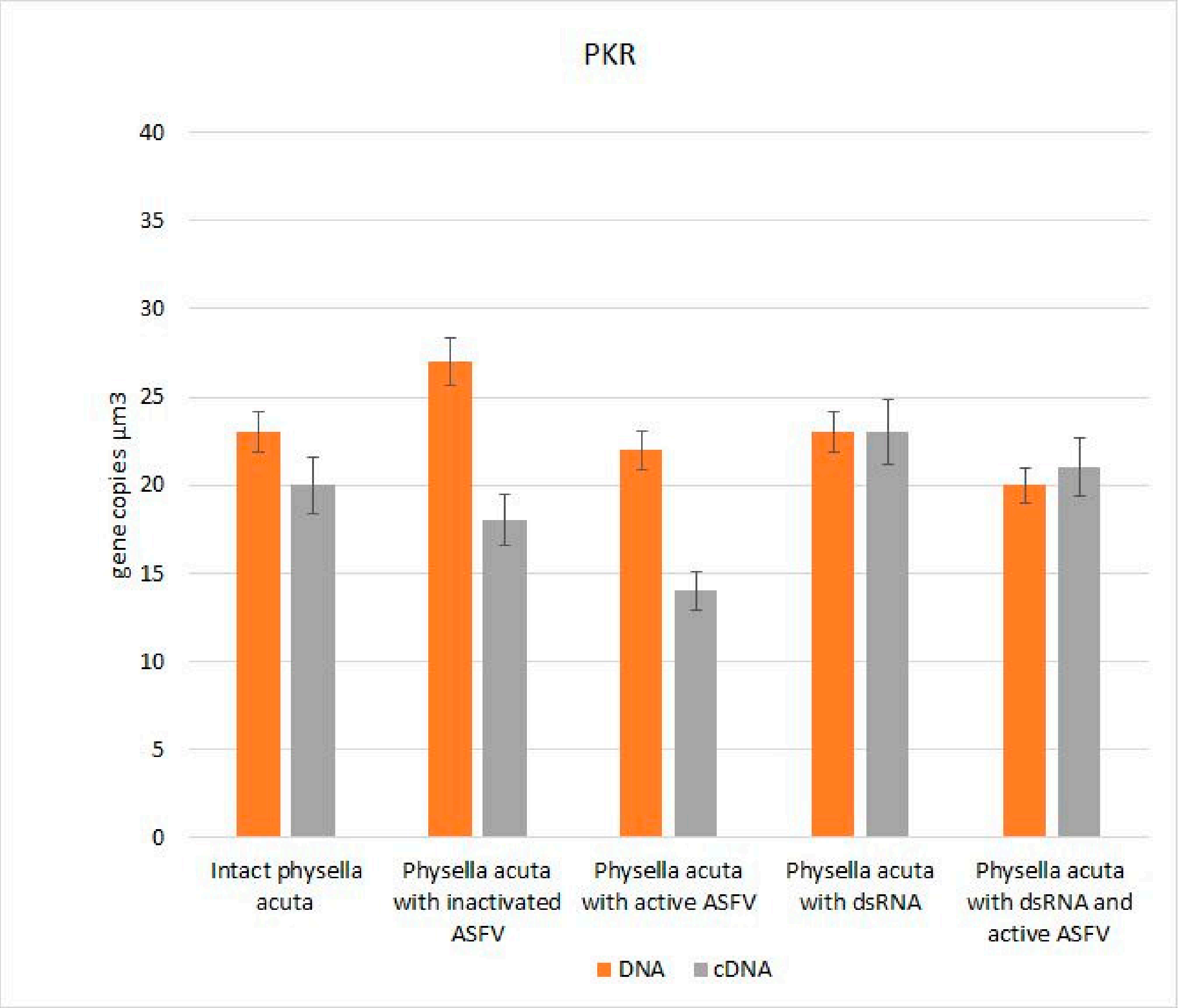

As follows from Figure S4, dsRNA does not activate the transcription of the PKR-like gene in the *Physella acuta* snail.
